# Is underweight associated with poorer diet, nutrient status, bone and cardiometabolic health, and school performance in Danish 8-11-year-olds?

**DOI:** 10.1007/s00394-024-03528-2

**Published:** 2024-11-14

**Authors:** Anne V. Aurup, Katrine Strandberg-Larsen, Rikke Andersen, Anja Biltoft-Jensen, Lotte Lauritzen, Camilla T. Damsgaard

**Affiliations:** 1 Department of Nutrition, Exercise and Sports, University of Copenhagen, Frederiksberg C, Denmark; 2 Department of Public Health, University of Copenhagen, Copenhagen K, Denmark; 3 National Food Institute, Technical University of Denmark, Kgs. Lyngby, Denmark

**Keywords:** Children, Thinness, Low BMI, Dietary intake, Nutrition, Biomarkers

## Abstract

**Purpose:**

Underweight, i.e. low body mass index for age and sex, may indicate undernutrition, but despite high prevalence, this aspect is largely overlooked in children in high-income countries. We explored if dietary intake, nutrient status, body composition, bone mineralization, cardiometabolic markers and school performance differed in schoolchildren with underweight compared to normal- and overweight.

**Methods:**

We used cross-sectional data from 815 Danish 8-11-year-old children collected in 2011. Intake of foods, macronutrients and key micronutrients (vitamin D, vitamin B12, calcium, iron, zinc and selenium) was assessed by 7-day dietary records. Measurements included anthropometry, dual-energy X-ray absorptiometry and tests of attention and reading skills. Fasting blood samples were analyzed for biomarkers of iron, long-chain n-3 fatty acids and vitamin D status as well as blood lipids, insulin and growth markers.

**Results:**

Eighty-three (10.2%) children had underweight and were shown to have a lower intake of energy, red meat, protein and zinc and higher intake of added sugar than children with normal- and overweight. They also had higher fish intake relative to overweight, but blood biomarkers did not differ between groups. Children with underweight had lower fat percent and bone mineralization compared to peers with normalweight, but apart from lower insulin, they did not differ in overall cardiometabolic health or school performance.

**Conclusion:**

Although we found some differences in diet, there were no considerable differences in nutrient status, cardiometabolic health or school performance between children with underweight and their normalweight peers. However, the lower bone mineralization is a concern and needs further investigation.

**Supplementary Information:**

The online version contains supplementary material available at 10.1007/s00394-024-03528-2.

## Introduction

Childhood underweight, also known as thinness, is used to denote low body mass index (BMI) in children relative to sex and age [1] and may be a marker of undernutrition. Childhood underweight is prevalent in low-income countries where it is linked to poverty, malnutrition, increased morbidity and mortality [2] and poor cognitive development [3]. However, underweight in childhood is also common in high-income countries, but is largely understudied, due to an unequivocal focus on childhood overweight. Thus, it is unknown whether it is a public health problem that needs to be prevented and what constitutes a healthy weight in childhood.

In Denmark, approximately 8% of school-aged children have underweight [4] and rates of 14%, 6% and 5% have been reported among 6–9 year-old children in France [5], Australia [6] and Portugal [7], respectively. Moreover, a meta-analysis showed that childhood underweight has increased from 7 to 9% in Europe over the last 10–15 years [8] and among children measured in the 1990s and 2000s, a recent Danish study found that underweight was as prevalent or even more prevalent than childhood obesity [9]. It is well-known that childhood overweight is associated with lower parental education and social class in high-income settings, but whether this is also the case for underweight or if underweight may even be linked with socioeconomic advantage and healthy lifestyle is unknown, as results are inconsistent [10–12].

Childhood underweight may be a result of inadequate energy and nutrient intake. Inadequate food and energy consumption increases the risk of deficiencies in important nutrients such as iron, long-chain n-3 polyunsaturated fatty acids (n-3 LCPUFA) and vitamin D, which are generally low in European children [13]. One cross-sectional study showed that underweight was linked to a lower adherence to the traditional Mediterranean diet among Greek children [14], while it was associated with a more regular meal pattern in Australian children [10]. However, we have little knowledge about the overall diet quality of children with underweight in high-income countries and to our knowledge, no study has investigated this combined with nutritional status assessed by biomarkers of nutrients such as iron, n-3 LCPUFA and vitamin D. These nutrients are important for cognitive function [15, 16], growth and bone health [17] as well as cardiometabolic health [18]. Studies from low-income countries show that underweight and undernutrition, particularly inadequate intakes of iron and n-3 LCPUFA impair children’s cognitive development and school performance [15, 19, 20]. However, the evidence among children with underweight in high-income settings is scarce.

Optimal bone mineralization during childhood and adolescence is an important determinant of adult bone health and osteoporosis and fractures later in life [21]. Higher BMI has been associated with higher bone mineral density (BMD) in children, likely due to the increased mechanical load on the weight bearing bones [22], and children with underweight may therefore have lower bone mineralization. Underweight has been linked to lower BMD in women [23] and adolescents with anorexia nervosa [22]. Moreover, overweight and obesity has been associated with increased risk of hypertension, dyslipidemia and high insulin in childhood [24], which all increase the risk of cardiovascular disease and type 2 diabetes [25]. In line with this, a few studies indicate that children and adolescents with underweight have the most favorable cardiometabolic risk profiles [26, 27].

In the present study, we aimed to investigate if childhood underweight constitutes a health concern in a high-income setting using cross-sectional data on 815 Danish 8-11-year-olds. We explored if children with underweight differed from those with normal- and overweight with regards to dietary intake, biomarkers of iron, n-3 LCPUFA and vitamin D status, body composition, bone mineralization and biomarkers of growth and bone formation, cardiometabolic markers and school performance.

## Materials and methods

### Study design and participants

This study used baseline data from a cluster-randomised, controlled, cross-over trial (the Optimal Well-Being, Development and Health for Danish Children through a Healthy New Nordic Diet (OPUS) School Meal Study) that was collected during August-November 2011. The study included 8-11-year-old children from forty-six classes at nine Danish municipal schools in the eastern part of Denmark [28]. All children in the classes were invited to participate in the study, but children were excluded if they had diseases or conditions that might interact with measurements, severe food allergies, or participated in other studies involving radiation or blood sampling [28]. The participants in OPUS were representative with respect to parental educational attainment and proportion of immigrants/descendants among families in Denmark [29]. The study design and school recruitment have been described in detail previously [28, 30]. A total of 834 children (82% of invited children) were enrolled. In the present study, we included the 815 children (98% of the original study population) who had available baseline data on age, sex, bodyweight and height (Supplementary Fig. 1).

### Measurements

#### Questionnaires on sociodemographics and puberty

At baseline, each family underwent a 2-hour in-person background interview including sociodemographics and puberty [28]. Socioeconomic status was evaluated as the highest parental education level in the household categorized into four categories based on years of schooling: ≤10 y, 11–12 y, 13–16 y & ≥17 y. Children were defined as immigrants or descendants if all grandparents and $\:\ge\:1$ parent were reported to be born outside of Denmark. In addition, the study staff categorized each child as ‘Caucasian’ or ‘non-Caucasian’ based on physical appearance and statement by the child in a separate variable [31].

Pubertal status was self-evaluated by the children with help from the parents based on visual depictions of pubic hair in boys and breast development in girls (Tanner stages) as validated by Morris and Udry [32]. In analysis, the variable was dichotomized as ‘entered puberty’ (stage 2–5) and ‘did not enter puberty’ (stage 1). Few children (7.0%) were above Tanner stage 2.

#### Dietary intake

With help from their parents, children recorded their daily intake of food and beverages during seven consecutive days using the validated interactive Web-based Dietary Assessment Software for Children (WebDASC) developed for the OPUS study [33]. Portion sizes were estimated by selecting the closest portion size among four different images, and individual energy and nutrient intakes were calculated using the General Intake Estimation System software developed at the National Food Institute, Technical University of Denmark [34] and given as an average of the recorded days. We excluded children from the analysis if they under- (*n* = 70) or over-reported (*n* = 14) their energy intake, as this can bias analysis of dietary intake. Children were classified as under- or over-reporters based on the ratio of reported energy intake and their calculated basal metabolic rate [35]. Under- and over-reporting were defined in accordance with the Goldberg cut-offs suggested by Black [36], ≤ 1.05 and ≥ 2.28, respectively, as previously reported [37]. Under-reporting of energy intake was more common among children with overweight (28.3%), compared to normalweight (5.1%) and underweight (11.3%).

The present study included data on total energy intake, intake of macronutrients and key micronutrients (vitamin D, vitamin B12, calcium, iron, zinc and selenium), as well as intake of five important food groups (vegetables, fruits, red meat, poultry and fish). For the micronutrients, we used estimated intake per 10 MJ, nutrient density, and calculated the proportion of children in each weight group with intakes below the average requirement (AR) as defined in the newly published Nordic Nutrition Recommendations 2023 [38]. Intake below the AR is associated with an increased probability of inadequacy [38]. We used sex- and age-specific AR values for the majority of the investigated micronutrients, but used so-called provisional AR values for vitamin B12 and selenium as AR values were not available for these nutrients [38].

#### Anthropometry, body composition and blood pressure

Clinical examinations and blood sampling were performed in a mobile laboratory at the schools. Except for body composition, all measurements were performed in the morning following an overnight fast. Using a stadiometer, height was measured barefoot to the nearest 0.1 cm with the head in the Frankfort horizontal position. Height was measured three times and the mean was used in this study. Bodyweight was measured wearing underwear or light clothing to the nearest 0.1 kg on a digital scale. BMI was calculated as the ratio of bodyweight to the square of height (kg/m^2^) and BMI z-scores were calculated based on the WHO Growth Reference from 2007 [39]. We categorized weight status as underweight, normalweight and overweight (incl. obesity) according to the age- and sex-specific cut-offs defined by centiles passing through a BMI of 18.5 and 25 kg/m^2^ at the age of 18, proposed by Cole and Lobstein [40] and the International Obesity Task Force (IOTF) classification [41]. Underweight was further subdivided into grade 3, grade 2 and grade 1 corresponding to BMI cut-offs of 16, 17 and 18.5 kg/m^2^ at the age of 18 [1].

Bone health and body composition was measured by dual-energy X-ray absorptiometry (DXA) using a Lunar Prodigy Pro SW scanner (GE Healthcare). Prior to the scan, most of the children had a standardized breakfast. Whole-body fat mass and fat-free mass as well as total-body-less-head BMD, bone mineral content (BMC), and bone area (BA) were derived from the DXA scans. Fat mass index and fat-free mass index were calculated by dividing fat mass and fat free mass (kg) with the square of height (m^2^), respectively. After a 10 min rest, blood pressure and heart rate were measured in the supine position with an automated device (UA-787 Plus; A&D Medical). Measurements were performed three times, and the mean of the last two measurements was used in this study.

#### Blood sampling and analysis

A 35 ml fasting venous blood sample was drawn from the forearm, processed as previously described [30, 31], and stored at -80℃ until analysis. Whole-blood haemoglobin was analyzed immediately after sampling using a Hemocue Hb 201 analyser (HemoCue Danmark, Denmark) [42]. Whole-blood EPA + DHA, as a biomarker of fish and n-3 LCPUFA intake, was measured by high-throughput gas chromatography, as previously described [30]. EPA + DHA is given as wt: wt% of the total fatty acids. Serum 25-hydroxyvitamin D (25(OH)D) was measured by immunoassay, as previously described [31], and used as biomarker of vitamin D status. Whole-blood haemoglobin and serum ferritin were analyzed, as previously described [42], and used as biomarkers of iron status and stores, respectively. Anemia was defined as haemoglobin < 7 mmol/L and depleted iron stores as serum ferritin < 15 µg/L. Plasma insulin-like growth factor-1 (IGF-1) was assessed as biomarker of growth and plasma osteocalcin as well as serum intact parathyroid hormone (iPTH) were used as biomarkers of bone formation and remodeling and together with serum insulin these markers were analyzed using automated immunoassays, as previously described [29, 43]. High density lipoprotein (HDL) cholesterol and triacylglycerol (TAG) were measured by automated assays and low density lipoprotein (LDL) cholesterol was calculated using Friedewald’s equation [44].

#### School performance

During the same week as the assessment of dietary intake, attention and reading skills were assessed as measures of school performance, as previously described [45]. The d2 Test of Attention involved a simple paper-and-pencil crossing-out task with 14 lines each with 47 letters that were processed under time pressure. The test was administered by the project staff and the children were instructed to mark as many target items as possible within 20 s per line. The outcomes from the d2 Test were processing speed defined as the total number of characters processed, the percentage of total errors as an indicator of inaccuracy as well as indicators of inattention and impulsivity defined by percentage of omission- and commission errors, respectively.

To assess reading skills, the teachers administered the Danish standard test ‘The Sentence Reading Test 2’ [46]. This test consisted of 27 drawings each accompanied by four sentences, and the children had to evaluate for each sentence whether it matched the associated drawing with the sentences gradually becoming longer and more complex. The children were given eight minutes to evaluate as many sentences as possible. Reading speed was defined by the total number of sentences read, irrespective of whether the sentences were correct or not, and reading proficiency was defined as the percentage of correctly read sentences.

### Statistical analysis

Results are presented for each weight group and include means and standard deviations (SD) for symmetrically distributed variables, medians and interquartile range (IQR) for skewed variables, and n (%) for categorical variables. Weight group differences in characteristics and outcomes were tested with one-way ANOVA, Kruskal-Wallis test or Pearson’s chi-square test, as appropriate. Post hoc tests with pairwise comparisons between weight groups were conducted using Tukey’s method or Dunn’s test with Sidák adjustment, as appropriate, and accounting for multiple testing. If the crude analysis showed any differences between children with underweight and the two other groups in outcomes related to dietary intake, nutrient status, bone health and growth, cardiometabolic health or school performance, we furthermore compared children with underweight to normal- and overweight in a multivariable analysis to adjust for potential confounders. All the employed linear mixed-effects models included class and school as random effects to account for dependency structures in the study population and weight group as fixed effect. Additionally, the models included age (continuous), sex, height (continuous) and parental education as potential confounders. Height was however omitted as covariate in the adjusted analysis of IGF-I and insulin to avoid overadjustment caused by the effect they have on growth and body composition in children [47–49]. We conducted a supplementary analysis with BMI as a continuous variable, if the results of the adjusted analysis indicated an association across the whole BMI-range. These analysis were performed by linear mixed-effects models that included age (continuous), sex, height (continuous) and parental education as fixed effects and class and school as random effects in the same way as described for the analysis across the three weight groups. Model assumptions were checked by residual plots and QQ plots. If necessary, outcomes were ln-transformed before analysis and estimates back-transformed to their original scale using the method by Laursen et al. [50].

As some of the children did not have a weekly intake of fish (*n* = 176), we calculated the percentage of children with an average fish intake of > 0 g/d, so-called eaters, in each weight group and restricted the analysis of median intake of fish to these children (*n* = 532). Similar, children who did not eat fish were excluded in the adjusted analysis of fish intake. To address this restriction, we examined the association between having any weekly intake of fish (yes / no) and weight group. This was done both in a crude unadjusted generalized linear model and a generalized linear mixed-effects model with the same adjustment as the described above, using the logit link function. Statistical analyses were conducted using the software STATA V.14.2 and R V.4.3.0. Significance was defined as *P* < 0.05.

## Results

### Children’s characteristics

Among the 815 included children, 83 (10.2%) had underweight of mainly grade 1 (9.1%) and 13.5% had overweight (Supplementary Table 1), as previously reported [29, 51]. There were no sex differences in the prevalence of underweight, normalweight and overweight (Supplementary Table 1).

Apart from having lower bodyweight, BMI and BMI z-score, children with underweight were shorter, less likely to have entered puberty and to be immigrants/descendants and more likely to have parents with long education, especially compared to children with overweight (Table 1). They also had lower fat mass- and fat free mass index as well as fat percent than children with normalweight (Table 1). There were no differences in age or ethnicity across weight groups.

**Table 1:** Characteristics of the study population according to weight group

	Underweight (*n* = 83)	Normalweight (*n* = 622)	Overweight/obesity (*n* = 110)	*P* ^†^
Sex, % girls	54.2	46.5	49.1	0.39
Age, y	10.0 (0.6)	9.9 (0.6)	10.1 (0.6)	0.06
Height, cm	140.9 (6.8)^[a]^	141.9 (6.9)^[a]^	146.7 (7.0)^[b]^	< 0.001
Bodyweight, kg	27.9 (25.5–29.4)^[a]^	33.5 (30.3–37.3)^[b]^	46.3 (42.3–50.0)^[c]^	< 0.001
BMI, kg/m^2^	14.2 (13.7–14.3)^[a]^	16.6 (15.6–17.8)^[b]^	21.3 (20.6–22.6)^[c]^	< 0.001
BMI-for-age z-score^**‡**^	-1.7 (0.4)	0.1 (0.7)	1.9 (0.5)	< 0.001
Fat mass index, kg/m^2^	2.1 (1.6–2.3)^[a]^	3.6 (2.6–4.6)^[b]^	7.7 (6.9–8.4)^[c]^	< 0.001
Fat free mass index, kg/m^2^	11.3 (0.6)^[a]^	12.4 (0.9)^[b]^	13.1 (1.1)^[c]^	< 0.001
Fat percent, %	14.6 (11.8–16.6)^[a]^	21.8 (16.2–26.8)^[b]^	35.8 (33.2–39.1)^[c]^	< 0.001
Pubertal status^**§,¶**^, % entered puberty	27.2^[a]^	32.6^[a]^	50.5^[b]^	< 0.001
Parental education level^**¶**^, %				
≤10 y	0	5	16.4	< 0.001
11–12 y	34.9	33.6	40	
13–16 y	36.1	38.9	36.4	
≥17.y	28.9^[a]^	22.5^[a]^	7.3^[b]^	
Child ethnicity (racial)^**††**^, n (%)				
Caucasian	77 (92.8)	583 (94.2)	106 (96.4)	0.53
Non-Caucasian	6 (7.2)	36 (5.8)	4 (3.6)	
Immigrant/descendant^**‡‡**^, n (%)				
No	78 (94.0)	558 (89.9)	84 (76.4)	< 0.001
Yes	5 (6.0)^[a]^	63 (10.1)^[a]^	26 (23.6)^[b]^	

BMI body mass index

The table shows means and standard deviations for symmetrically distributed variables, medians and interquartile range for skewed variables and n (%) for categorical variables

^**†**^Differences between weight groups were determined by one-way ANOVA, Kruskal-Wallis test or Pearson’s chi-square test, as appropriate. Post hoc tests involved pairwise comparisons between weight groups and were conducted using Tukey’s method or Dunn’s test with Sidák adjustment

^**‡**^based on the WHO Growth Reference from 2007 [39]

^**§**^Pubertal status was self-evaluated using Tanner stages (visual depictions) with stages 2–5 categorized as ‘entered puberty’ and stage 1 as ‘not entered puberty’

^**¶**^*n* = 81 underweight; *n* = 602 normalweight; *n* = 105 overweight/obesity

^**††**^*n* = 619 normalweight

^**‡‡**^*n* = 621 normalweight

^[a], [b], [c]^Values with dissimilar superscript letters were different between groups (*P* < 0.05)

### Dietary intake and nutritional biomarkers

As expected, the crude analysis showed that children with underweight had lower energy intake compared to children with normal- and overweight (Table 2). Underweight was also associated with lower intake of protein and red meat and higher intake of added sugar compared to the two other weight groups, and with higher fish intake compared to overweight (Table 2). However, there were no differences between weight groups in the dichotomized (yes / no) analysis of any fish intake (Supplementary Table 2) or in whole-blood EPA + DHA (Table 2).

Overall, the diet of children with underweight did not differ in micronutrient density, except for a lower content of zinc (Table 2). However, the proportion of children with intake below the AR did not differ for zinc, but the frequency of a low vitamin B12 intake was slightly higher in the children with underweight (Supplementary Table 3). As shown previously [31, 37], vitamin D intakes were generally insufficient, but did not differ between weight groups (Table 2 and Supplementary Table 3). The proportions of children with calcium, iron and selenium intakes below the AR were also high (between one fifth and half), but with no differences between groups (Supplementary Table 3). No other weight group differences in dietary intake or nutritional biomarkers were found (Table 2).

**Table 2:** Dietary intake of the children according to weight group

	Underweight	Normalweight	Overweight/obesity	
	(*n* = 69)	(*n* = 563)	(*n* = 76)	*P* ^†^
**Dietary intake**				
Energy, kJ/d	7185 (6461–8148)^[a]^	7679 (6789–8708)^[b]^	7757 (6616–9000)^[b]^	0.013
Protein, E%	14.6 (2.2)^[a]^	15.3 (2.0)^[b]^	15.7 (2.1)^[b]^	0.006
Fat, E%	32 (4)	32 (4)	32 (4)	0.1
SFA, E%	12 (2)	12 (2)	12 (2)	0.96
MUFA, E%	11 (2)	11 (2)	11 (2)	0.54
PUFA, E%	5 (4–5)	5 (4–5)	5 (4–5)	0.71
n-6 PUFA, E%	4 (4–4)	4 (3–4)	4 (3–4)	0.89
n-3 PUFA, E%	1 (1–1)	1 (1–1)	1 (1–1)	0.29
Carbohydrate, E%	53 (5)	53 (5)	52 (4)	0.41
Added sugar, E%	12 (9–15)^[a]^	11 (8–14)^[b]^	10 (8–13)^[b]^	0.023
Dietary fiber, g/10 MJ	23 (19–27)	23 (20–27)	24 (20–26)	0.84
Vitamin D, µg/10 MJ	2.3 (1.9-4.0)	2.5 (1.8–3.5)	2.1 (1.8–2.9)	0.18
Vitamin B12, µg/10 MJ	5.9 (4.5–7.7)	5.8 (4.5–7.6)	5.8 (4.7–7.1)	0.93
Calcium, mg/10 MJ	1191 (966–1351)	1186 (984–1373)	1178 (959–1437)	0.96
Iron, mg/10 MJ	11.0 (10.0-12.1)	11.3 (10.2–12.4)	11.7 (10.3–12.6)	0.13
Zinc, mg/10 MJ	12.1 (10.4–13.2)^[a]^	12.7 (11.3–13.7)^[b]^	13.1 (12.0-14.4)^[c]^	0.001
Selenium, µg/10 MJ	48 (40–57)	49 (43–58)	48 (42–56)	0.41
Vegetables & vegetable products, g/10 MJ	162 (99–226)	172 (117–228)	163 (125–230)	0.44
Fruits & fruit products, g/10 MJ	160 (101–232)	169 (101–252)	181 (101–235)	0.81
Red meat & meat products, g/10 MJ	108 (63–132)^[a]^	119 (87–155)^[b]^	142 (102–171)^[c]^	< 0.001
Poultry & poultry products, g/10 MJ	19 (4–39)	21 (6–46)	24 (9–41)	0.77
Eaters of fish & fish products^**‡**^, n (%)	56 (81.2)	425 (75.5)	51 (67.1)	0.14
Fish & fish products^**§**^, g/10 MJ	27 (11–51)^[a]^	26 (12–43)^[a]^	16 (6–36)^[b]^	0.049
**Nutritional biomarkers** ^**¶**^				
Serum 25(OH)D, nmol/L	62.1 (20.6)	61.7 (18.0)	59.8 (18.0)	0.7
EPA + DHA^**††**^, wt: wt%	3.57 (0.94)	3.62 (0.97)	3.48 (0.96)	0.49
Haemoglobin, mmol/L	8.2 (0.5)	8.2 (0.5)	8.3 (0.5)	0.48
Serum ferritin, µg/L	40 (30–51)	40 (30–53)	42 (30–56)	0.77

25(OH)D 25-hydroxyvitamin D, E% percentage of energy intake, MJ megajoule

The table shows means and standard deviations for symmetrically distributed variables, medians and interquartile range for skewed variables and n (%) for categorical variables

^**†**^Differences between weight groups were determined by one-way ANOVA, Kruskal-Wallis test or Pearson’s chi-square test, as appropriate. Post hoc tests involved pairwise comparisons between weight groups and were conducted using Tukey’s method or Dunn’s test with Sidák adjustment

^**‡**^Eaters are defined as children with fish intake > 0 g/d

^**§**^Based on eaters only, i.e. children with fish intake > 0 g/d. *n* = 56 underweight; *n* = 425 normalweight; *n* = 51 overweight/obesity

^**¶**^*n* = 53–67 underweight; *n* = 440–548 normalweight; *n* = 56–75 overweight/obesity

^**††**^Values are given as wt: wt% of all whole-blood fatty acids

^[a], [b], [c]^Values with dissimilar superscript letters were different between groups (*P* < 0.05)

Adjustment for age, sex, height and parental education removed the difference in total energy intake between underweight and overweight, but did not change any of the other weight group differences found in Table 2 (Supplementary Table 4).

### Bone health & growth and cardiometabolic markers

Children with underweight had lower BMD, BMC and BA than the other weight groups (Table 3). Underweight was also associated with lower circulation concentrations of the growth factor IGF-1 as well as insulin compared to normal- and overweight (Table 3). Children with underweight had slightly higher heart rate than their normalweight peers and both under- and normalweight children had lower systolic blood pressure than children with overweight (Table 3). Similarly, fasting blood lipids did not differ between children in the two lower BMI categories, but both these groups had higher HDL cholesterol and lower LDL cholesterol and TAG than children with overweight (Table 3).

**Table 3:** Bone health, growth and markers of cardiometabolic health according to weight group

	Underweight (*n* = 83)	Normalweight (*n* = 620)	Overweight/obesity (*n* = 110)	*P* ^†^
**Bone health & growth factors** ^**‡**^				
BMD, g/cm^3^	0.73 (0.05)^[a]^	0.78 (0.05)^[b]^	0.85 (0.06)^[c]^	< 0.001
BMC, g	749 (668–858)^[a]^	927 (808–1042)^[b]^	1170 (1019–1269)^[c]^	< 0.001
BA, cm^2^	1039 (960–1141)^[a]^	1177 (1068–1285) ^[b]^	1366 (1258–1476) ^[c]^	< 0.001
iPTH, pmol/L	3.2 (2.3–3.9)	3.1 (2.3–4.1)	3.3 (2.5–4.2)	0.31
Osteocalcin, ng/mL	30 (24–36)	29 (23–36)	32 (25–38)	0.20
IGF-I, ng/mL	169 (127–207)^[a]^	193 (159–233)^[b]^	213 (179–267)^[c]^	< 0.001
**Cardiometabolic markers** ^**§**^				
Systolic blood pressure, mmHg	106 (8)^[a]^	107 (8)^[a]^	110 (9)^[b]^	0.005
Diastolic blood pressure, mmHg	68 (6)^[a, b]^	68 (6)^[a]^	70 (7)^[b]^	0.019
Heart rate, beats/min	81 (12)^[a]^	78 (11)^[b]^	79 (13)^[a, b]^	0.016
Insulin, pmol/L	35 (26–43)^[a]^	41 (31–56)^[b]^	62 (48–87)^[c]^	< 0.001
LDL cholesterol, nmol/L	2.3 (0.6)^[a]^	2.3 (0.6)^[a]^	2.5 (0.6)^[b]^	< 0.001
HDL cholesterol, nmol/L	1.5 (0.3)^[a]^	1.5 (0.3)^[a]^	1.3 (0.3)^[b]^	< 0.001
TAG, mmol/L	0.6 (0.5–0.7)^[a]^	0.6 (0.5–0.8)^[a]^	0.7 (0.6–1.1)^[b]^	< 0.001

BMD bone mineral density, BMC bone mineral content, BA bone area, iPTH intact parathyroid hormone, IGF-I insulin-like growth factor-1, TAG triacylglycerol

The table shows means and standard deviations for symmetrically distributed variables and medians and interquartile range for skewed variables

^**†**^Differences between weight groups were determined by one-way ANOVA or Kruskal-Wallis test, as appropriate. Post hoc tests involved pairwise comparisons between weight groups and were conducted using Tukey’s method or Dunn’s test with Sidák adjustment

^**‡**^*n* = 73–83 underweight; *n* = 558–619 normalweight; *n* = 98–110 overweight/obesity

^**§**^*n* = 80–83 underweight; *n* = 602–620 normalweight; *n* = 109–110 overweight/obesity

^[a], [b], [c]^Values with dissimilar superscript letters were different between groups (*P* < 0.05)

Adjustment for age, sex, height and parental education rendered the difference in systolic blood pressure between underweight and overweight non-significant, but did not change any of the other weight group differences found in Table 3 (Supplementary Table 4).

### School performance

Children with underweight did not differ from the other weight groups in processing speed or percentages of errors made in the d2 Test of Attention (Table 4). Likewise, there were no differences in reading speed or reading proficiency from the sentence reading test between weight groups (Table 4).

**Table 4:** School performance assessed by attention and reading skills according to weight group

	Underweight (*n* = 75)	Normalweight (*n* = 594)	Overweight/obesity (*n* = 104)	*P* ^†^
**The d2 Test of attention** ^**‡**^				
Processing speed^**§**^	336 (62)	330 (57)	330 (54)	0.72
Total error %	1.9 (0.9–3.7)	2.0 (1.0-3.4)	2.3 (1.4–4.2)	0.08
Omission error %	1.5 (0.7-3.0)	1.6 (0.8–2.9)	1.8 (1.1–3.2)	0.20
Commission error %	0.3 (0.0-0.6)	0.3 (0.0-0.5)	0.3 (0.0-0.9)	0.18
**Sentence reading test** ^**¶**^				
Reading speed^**††**^	56 (16)	56 (16)	54 (15)	0.43
Reading proficiency^**‡‡**^	97.6 (93.1–100)	96.9 (93.0-98.6)	95.8 (92.3–98.3)	0.08

The table shows means and standard deviations for symmetrically distributed variables and medians and interquartile range for skewed variables

^**†**^Differences between weight groups were determined by one-way ANOVA or Kruskal-Wallis test, as appropriate

^**‡**^*n* = 74 underweight; *n* = 569 normalweight

^**§**^The total number of characters processed during the test

^**¶**^*n* = 593–594 normalweight; *n* = 102 overweight/obesity

^**††**^The total number of sentences read irrespective of whether the sentences were correct or not

^**‡‡**^The percentage of sentences correctly read

### Continuous analysis of BMI

The observed differences in the intake of protein, red meat, zinc and added sugar between children with underweight and the other weight groups were also supported by continuous linear regression analysis across the entire BMI range (Table 5). The associations between weight groups and BMD, IGF-I and insulin levels were furthermore supported by continuous linear regression analysis, which were estimated to increase with 0.01 g/cm^3^ (95% CI (0.01,0.01); *P* < 0.001) in BMD, 7 ng/mL (95% CI (5,9); *P* < 0.001) in IGF-I and 4 pmol/L (95% CI (3,4); *P* < 0.001) in insulin levels for each one unit BMI increment (Table 5).

**Table 5:** Linear regression analysis of associations between BMI and specific outcomes

	BMI		
**Dependent variable**	*n*	$\:\beta\:$ [95% CI]	*P*
Intake of protein, E%	707	0.1 [0.0;0.2]	0.001
Intake of added sugar, E%	707	-0.3[-0.4;-0.1]	0.001
Intake of Zinc^**†**^, mg/10 MJ	707	0.1 [0.1;0.2]	< 0.001
Intake of meat & meat products^**†**^, g/10 MJ	707	4 [2;6]	< 0.001
BMD, g/cm^3^	809	0.01 [0.01;0.01]	< 0.001
IGF-I^**†,‡**^, ng/mL	773	7 [5;9]	< 0.001
Insulin^**†,‡**^, pmol/L	743	4 [3;4]	< 0.001

CI confidence interval, BMD bone mineral density, IGF-I insulin-like growth factor-1, E% percentage of energy intake, MJ megajoules

Data are presented as regression coefficients ($\:\beta\:$) with 95% confidence intervals (CI) and *P* values per unit increase in BMI derived from linear mixed-effects models adjusted for sex, age (continuous), height (continuous) and parental education level

^**†**^Dependent variable was ln-transformed to meet model assumptions and the table presents back-transformed estimates on the original scale based on the method by Laursen et al. [50]

^**‡**^The analysis did not include adjustment for height

## Discussion

In this study, children with underweight had lower intake of energy, protein and zinc and higher intake of added sugar. They also had lower red meat intake and higher reported fish intake, compared to those with overweight, which is in line with the Danish Official Dietary Guidelines [52], but with no differences in blood biomarkers. Importantly, children with underweight also had lower bone mineralization, which could be a concern in relation to bone health later in life. Apart from lower insulin, which may indicate a metabolic advantage or slower growth, the cardiometabolic profile and school performance of underweight children were similar to that of normalweight children.

The higher parental education, lower intake of red meat and higher reported intake of fish among underweight children in the present study may suggest that underweight in this context could be linked to a more healthy lifestyle and socioeconomic advantage, rather than deprivation, as seen in a large representative study from the UK [11]. However, we also found that underweight was associated with higher intake of added sugar, which does not indicate a healthier diet. The Danish National Dietary Surveys show that higher parental education and income is associated with better eating habits in children, including higher intakes of fish and vegetables [53], and the same pattern is seen in e.g. Germany [54] and the Netherlands [55]. In the present study, adjustment for variation in parental educational level across weight groups did not change the differences in dietary intake between groups, but it did render the difference in energy intake between underweight and overweight insignificant.

The difference observed in fish intake between underweight and overweight was not mirrored in whole blood EPA + DHA, which is a biomarker of fish and n-3 LCPUFA intake during the last weeks to months. It is possible that the self-reported fish intakes were particularly prone to social desirability bias among the children with underweight. Moreover, the lower meat intake was not clearly reflected in blood haemoglobin or in serum ferritin, a biomarker of iron stores, possibly because iron intake per kg did not differ (*data not shown*) or since intestinal iron absorption increases with lower status to maintain adequacy [56]. Furthermore, very few children had anemia (*n* = 2) and depleted iron stores (*n* = 10) in this study. In line with the lower meat intake, zinc intake was also lower among the underweight children. However, we found no differences in the proportion of children with zinc intake below the AR. In contrast, children with underweight were more likely to have vitamin B12 intake below the AR. Of note, generally up to half of the children had selenium intake below the AR. This is a known concern in the Nordic countries related to low soil contents.

We found that children with underweight had considerably lower body fat (index and percent) and only a slightly lower lean mass index than children in the normal and higher weight categories. In addition, Freedman et al. [57] showed that BMI differences among children with underweight were mainly caused by differences in fat-free mass index rather than fat mass index. It is of concern, that underweight children had lower bone mineralization than children with normalweight, both with regards to BMC, BMD and BA. Similar, Kouda et al. [58] showed that, underweight in young Japanese children was associated with lower BMC, but also with lower fat free and fat mass later in childhood. Other studies have shown that very low bodyweight in adolescents with anorexia nervosa was associated with low bone mineralization [59, 60]. The lower bone status could be a direct consequence of the lower weight bearing, as higher load on the weight bearing bones as well as BMI in childhood is linked to higher bone density [22]. Bone mineral accretion during childhood is important for peak bone mass in adolescence, which is essential for adult bone health and prevention of osteoporosis [21]. Vitamin D intake and status also impact bone mineralization via its effect on calcium absorption [38]. As shown previously [31], vitamin D intakes were generally low, as seen in the overall Danish population due to limited food fortification [61]. However, vitamin D and calcium intakes did not differ between weight groups, so intake of these nutrients alone is unlikely to explain the differences in bone mineralization.

Underweight was also associated with lower serum concentrations of IGF-I. During childhood, growth hormone stimulates linear growth via hepatic secretion of IGF-I, which is a key regulator of bone metabolism [47], BMC [62] and a driver of linear growth [63]. IGF-I and fat mass both impact the onset of puberty, especially among girls [64]. However, despite lower IGF-I and fat mass among the underweight children in the present study, we could not detect differences in pubertal status among these children and those with normalweight. This could be because the underweight children in this study were mainly of grade 1, i.e. the mildest degree of underweight.

Childhood overweight and fatness is associated with higher fasting insulin [65, 66]. We found that children with underweight had lower serum insulin compared to the two other weight groups, which fits with the observed group differences in body composition, as insulin is an important anabolic hormone and growth factor that stimulates accretion of both lean and fat mass in childhood [48, 49]. However, as the present study was cross-sectional it was not possible to examine if the lower serum insulin reduced BMI and fat mass or vice versa. Lower fasting insulin could be linked to improved insulin sensitivity, but we did not find a consistent cardiometabolic advantage in the underweight children vs. their normalweight peers, as none of the other cardiometabolic markers were lower and heart rate was slightly higher.

There were no differences in attention or reading skills between children with underweight and the two other weight groups. However, Hjort and colleagues found lower school performance among children with underweight using data on math and reading skills [67]. Their results are also based on measurements from the OPUS study, but underweight was only linked to lower performance in analysis adjusting for lifestyle indicators, suggesting that healthy lifestyles in these families are important for the children’s performance in school [67]. Furthermore, among Canadian children, Bisset et al. [68] found lower teacher-reported academic performance among those that followed a low compared to normal BMI trajectory. Thus, more research is needed on school performance among children with underweight.

A major strength of the present study is the objective and golden standard measurements of the children’s height and weight, nutrient status, body composition, bone mineralization and cardiometabolic health. In addition, dietary intake was measured by 7-day dietary records using the same method as in the Danish National Dietary Surveys [69]. Moreover, the study had high participation rate (82%) and the study population have previously been demonstrated to be representative of Danish children in terms of parental education level and proportion of immigrants and descendants [29]. The limitations include the sample size and low number of children with underweight, especially of grade 2 and 3, which limits our ability to detect differences between the weight groups. Moreover, as the underweight children had mainly underweight of the mildest grade, the results from this study may therefore not be generalizable to populations of children with more extreme grades of underweight. However, the proportion of underweight children in this study corresponds well to the Danish national prevalence of underweight of approximately 8% among schoolchildren [4]. In this study, children recorded their dietary intake with parental support, which may have affected the data collected, as in particularly the oldest children might consume food and beverages outside the home without their parents’ knowledge, and maybe even foods they know their parents disapprove. Furthermore, we evaluated the children’s body size and categorized them into weight groups based on the widely used international age- and sex-specific BMI cut-offs [40]. However, there are several other metrics of underweight, so the findings in this study may not be directly comparable to studies using other definitions. In the present study, the cross-sectional design does not allow for examination of any temporal relationships or drawing conclusions about causality between the investigated variables. Finally, this study is of explorative purpose with a wide array of investigated outcomes, and thus involves possible change findings due to multiple testing. However, post hoc analysis between weight groups were adjusted for multiple comparisons.

## Conclusion

Children with underweight had lower intake of energy, red meat, protein and zinc and higher intake of added sugar than their normalweight peers, but did not differ in overall nutrient status in this high-income setting. Underweight was also associated with lower fat free- and especially fat mass index, but not with overall cardiometabolic profile or school performance. The lower bone mineralization seen in children with underweight is an area of concern and how it impacts peak bone mass and later risk of osteoporosis needs further investigation. Therefore, we suggest that future studies address whether childhood underweight shows tracking into late adolescence and adulthood and the potential impact of trajectories of childhood underweight on bone mineralization over the life span. Furthermore, we need more knowledge on the association between childhood underweight and risk of eating disorders and impaired mental health.

## Electronic supplementary material

Below is the link to the electronic supplementary material.

Supplementary Material 1

## Data Availability

Data described in the manuscript, code book, and analytic code will not be made available because data are not anonymized and due to the Danish legislation therefore considered as “personal data”.

## Abbreviations

AR: Average requirement
BA: Bone area
BMC: Bone mineral content
BMD: Bone mineral density
BMI: Body mass index
DXA: Dual energy X-ray absorptiometry
HDL cholesterol: High density lipoprotein cholesterol
IGF-1: Insulin-like growth factor-1.
IOTF: The International Obesity Task Force
iPTH: Intact parathyroid hormone
LDL cholesterol: Low density lipoprotein cholesterol
NNR2023: The Nordic Nutrition Recommendations 2023
n-3 LCPUFA: Long-chain n-3 polyunsaturated fatty acids (≤ 20 carbons and ≥ 3 double bonds)
TAG: Triacylglycerol
25(OH)D: 25-hydroxyvitamin D
