# Supplementary material for: Is underweight associated with poorer diet, nutrient status, bone and cardiometabolic health, and school performance in Danish 8-11-year-olds?
Source: Eur J Nutr. 2024 Nov 14;64(1):4. doi: 10.1007/s00394-024-03528-2 (PMC11564234; doi:10.1007/s00394-024-03528-2)
Supplement: Supplementary file 1 — Supplementary Material 1 [file 394_2024_3528_MOESM1_ESM.docx]

**ONLINE SUPPLEMENTARY INFORMATION - APPENDIX A**

**EUROPEAN JOURNAL OF NUTRITION**

**Is underweight associated with poorer diet, nutrient status, bone and cardiometabolic health, and school performance in Danish 8-11-year-olds?**

Anne V. Aurup^1,*^, Katrine Strandberg-Larsen^2^, Rikke Andersen^3^, Anja Biltoft-Jensen^3^, Lotte Lauritzen^1^ & Camilla T. Damsgaard^1^

^1^Department of Nutrition, Exercise and Sports, University of Copenhagen, Frederiksberg C, Denmark;

^2^Department of Public Health, University of Copenhagen, Copenhagen K, Denmark

^3^National Food Institute, Technical University of Denmark, Kgs. Lyngby, Denmark

*Corresponding author

***Corresponding Author Information**

Anne V. Aurup, [ava@nexs.ku.dk](mailto:ava@nexs.ku.dk).

**Supplementary Fig. 1** Participant flow chart


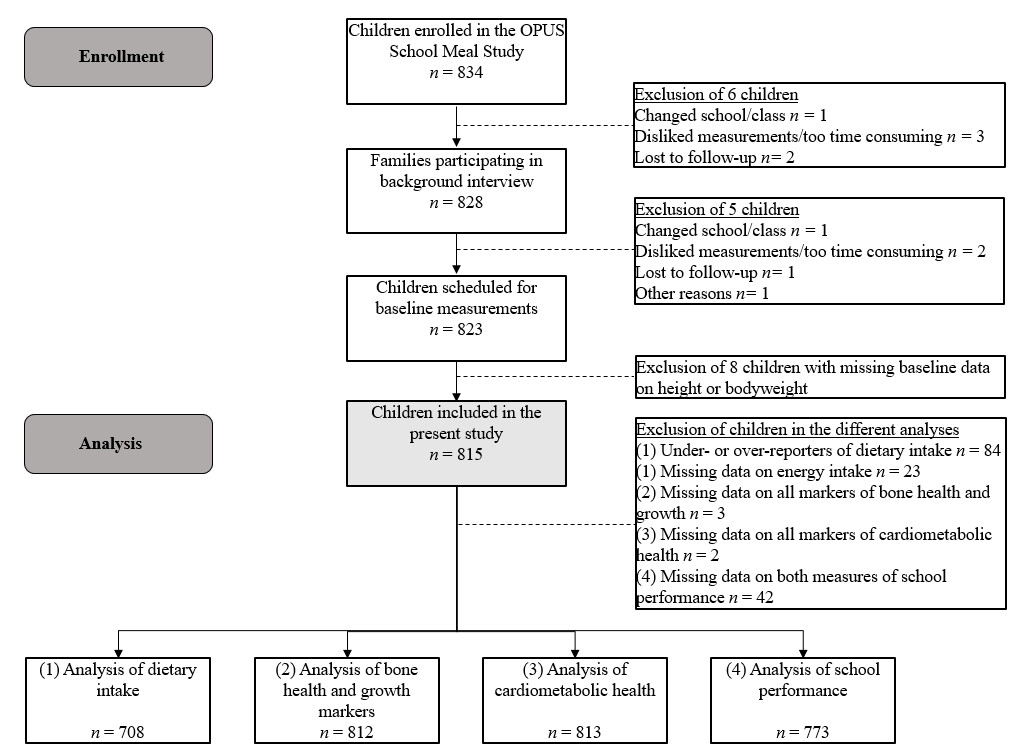


**Supplementary Table 1** Weight status of children in the study population

|  | All (*n* = 815) | Girls (*n* = 388) | Boys (*n* = 427) | *P***^†^** |
| --- | --- | --- | --- | --- |
| **Weight group^‡^, n (%)** |  |  |  |  |
| Underweight  *Grade 1* | 83 (10.2)  74 (9.1) | 45 (11.6)  40 (10.3) | 38 (8.9)  34 (8.0) | 0.39 |
| Normalweight | 622 (76.3) | 289 (74.5) | 333 (78.0) |  |
| Overweight incl. obesity | 110 (13.5) | 54 (13.9) | 56 (13.1) |  |
| *Obesity* | 16 (2.0) | 6 (1.5) | 10 (2.3) |  |

**^†^**Pearson’s chi-squared test conducted with weight group defined by underweight, normalweight and overweight/obesity
**^‡^**Weight group was categorized as underweight, normalweight, overweight and obesity according to the age- and sex specific cut-offs defined by centiles passing through a BMI of 18.5, 25 and 30 kg/m^2^ at the age of 18, proposed by Cole and Lobstein [1] and the International Obesity Task Force (IOTF) classification [2]. Underweight was further subdivided into grade 3, grade 2 and grade 1 corresponding to BMI cut-offs of 16, 17 and 18.5 kg/m^2^ at 18 years [3]. Due to small numbers only results for underweight grade 1 are presented in this table

**Supplementary Table 2** Crude and adjusted dichotomized analysis of any weekly fish intake (yes / no)

|  |  | **Crude analysis^†^** | | |  |  | **Adjusted analysis^‡^** | | |  |
| --- | --- | --- | --- | --- | --- | --- | --- | --- | --- | --- |
|  |  | UW vs. NW |  | UW vs. OW |  |  | UW vs. NW | UW vs. OW | |  |
|  | *n* | $OR$ [95% CI] | *P* | $OR$ [95% CI] | *P* | *n* | $OR$ [95% CI] | *P* | $OR$ [95% CI] | *P* |
| Any fish intake, yes | 708 | 1.40 [0.76;2.74] | 0.30 | 2.11 [0.99;4.67] | 0.06 | 707 | 1.41 [0.74;2.68] | 0.29 | 2.01 [0.90-4.46] | 0.09 |

UW underweight, NW normalweight, OW overweight/obesity, OR odds ratio, CI confidence interval
**^†^**Data are presented as odds ratios (OR) for any weekly intake of fish with 95% confidence intervals (CIs) and *P* values derived from unadjusted generalized linear
models with logit link function and weight group as independent variable
**^‡^**Data are presented as odds ratios (OR) for any weekly intake of fish with 95% confidence intervals (CI) and *P* values derived from generalized linear mixed-effects
models with logit link function and weight group, sex, age (continuous), height (continuous) and parental education as fixed effects and class and school as random effects

**Supplementary Table 3** The number and proportion of children in each weight group with intakes below the AR as defined by NNR2023 for selected micronutrients

|  | AR/provisional AR reference value**^†^** | Underweight (*n* = 69) | Normalweight (*n* = 563) | Overweight/obesity (*n* = 76) | *P***^‡^** |
| --- | --- | --- | --- | --- | --- |
| **Vitamins and minerals, n (%) < AR** |  |  |  |  |  |
| Vitamin D | 7.5 µg/d | 64 (92.8) | 531 (94.3) | 73 (96.1) | 0.69 |
| Vitamin B12**^§^** | ≤10 y: 2.0 µg/d ≥11 y girls: 2.8 µg/d ≥11 y boys: 2.6 µg/d | 7 (10.1)^[a]^ | 18 (3.2)^[b]^ | 2 (2.6)^[b]^ | 0.015 |
| Calcium | ≤10 y: 675 mg/d ≥11 y: 980 mg/d | 21 (30.4) | 106 (18.8) | 16 (21.1) | 0.08 |
|  |  |  |  |  |  |
| Iron | ≤10 y: 7 mg/d ≥11 y girls: 10 mg/d ≥11 y boys: 9 mg/d | 18 (26.1) | 109 (19.4) | 16 (21.1) | 0.41 |
|  |  |  |  |  |  |
| Zinc | ≤10 y: 6.4 mg/d ≥11 y girls: 9.0 mg/d ≥11 y boys: 9.2 mg/d | 9 (13.0) | 38 (6.8) | 4 (5.3) | 0.13 |
|  |  |  |  |  |  |
| Selenium**^3^** | ≤10 y: 35 µg/d ≥11 y: 50 µg/d | 36 (52.2) | 229 (40.7) | 32 (42.1) | 0.19 |

NNR2023 the Nordic Nutrition Recommendations 2023 [4], AR average requirement
**^†^**Only cut-offs for AR and provisional AR values relevant for the age range in this study are presented in the table
**^‡^**Pearson’s chi-square test
**^§^**Provisional AR values
^[a],[b],[c]^Values with dissimilar superscript letters were different between groups (*P* < 0.05)

**Supplementary Table 4** Crude and adjusted associations between weight group and dietary intake, bone health, growth and markers of cardiometabolic health

|  |  | | **Crude analysis^†^** | | | |  | | **Adjusted analysis^‡^** | | | | |
| --- | --- | --- | --- | --- | --- | --- | --- | --- | --- | --- | --- | --- | --- |
|  | UW vs. NW | | |  |  | UW vs. OW | | |  | UW vs. NW | | UW vs. OW | |
|  | *n* | $\beta$ [95% CI] | | | *P* | $\beta$ [95% CI] | | *P* | *n* | $\beta$ [95% CI] | *P* value | $\beta$ [95% CI] | *P* |
| **Dietary intake** |  |  | | |  |  | |  |  |  |  |  |  |
| Energy, kJ/d | 708 | -556 [-904;-208] | | | 0.002 | -578 [-1032;-125] | | 0.013 | 707 | -387 [-697;-77] | 0.015 | -243 [-657;171] | 0.25 |
| Protein, E% | 708 | -0.7 [-1.2;-0.2] | | | 0.007 | -1.0 [-1.7;-0.4] | | 0.002 | 707 | -0.7 [-1.2;-0.2] | 0.008 | -1.0 [-1.7;-0.4] | 0.003 |
| Added sugar, E% | 708 | 1.1 [0.04;2.2] | | | 0.042 | 1.8 [0.4;3.2] | | 0.011 | 707 | 1.2 [0.2;2.2] | 0.025 | 2.2 [0.8;3.5] | 0.002 |
| Zinc**^§^**, mg/10 MJ | 708 | -0.8 [-1.3;-0.3] | | | 0.002 | -1.3 [-1.9;-0.6] | | <0.001 | 707 | -0.8 [-1.2;-0.3] | 0.003 | -1.2 [-1.9;-0.6] | <0.001 |
| Meat & meat products**^§^**, g/10 MJ | 708 | -22 [-35;-9] | | | 0.001 | -39 [-56;-22] | | <0.001 | 707 | -21 [-34;-8] | 0.002 | -38 [-56;-21] | <0.001 |
| Fish & fish products**^§,††^**, g/10 MJ | 532 | 1 [-5;7] | | | 0.63 | 9 [1;18] | | 0.022 | 531 | 1 [-5;7] | 0.77 | 8 [0;17] | 0.05 |
| **Bone health & growth factors** |  |  | | |  |  | |  |  |  |  |  |  |
| BMC, g | 812 | -150 [-193;-109] | | | <0.001 | -394 [-446;-342] | | <0.001 | 809 | -128 [-151;-106] | <0.001 | -263 [-292;-233] | <0.001 |
| BMD, g/cm^3^ | 812 | -0.05 [-0.06;-0.04] | | | <0.001 | -0.11 [-0.13;-0.10] | | <0.001 | 809 | -0.05 [-0.06;-0.04] | <0.001 | -0.09 [-0.10;-0.07] | <0.001 |
| BA, cm^2^ | 812 | -121 [-158;-84] | | | <0.001 | -321 [-367;-275] | | <0.001 | 809 | -100 [-117;-83] | <0.001 | -199 [-220;-177] | <0.001 |
| IGF-I**^§,¶^**, ng/mL | 776 | -28 [-42;-13] | | | <0.001 | -60 [-78;-41] | | <0.001 | 773 | -33 [-47;-20] | <0.001 | -59 [-76;-42] | <0.001 |
| **Cardiometabolic markers** |  |  | | |  |  | |  |  |  |  |  |  |
| Systolic blood pressure, mmHg | 813 | -1.0 [-2.8;0.9] | | | 0.29 | -3.4 [-5.7;-1.1] | | 0.003 | 810 | -0.9 [-2.6;0.9] | 0.35 | -2.1 [-4.4;0.2] | 0.08 |
| Heart rate, beats/min | 813 | 4 [1;6] | | | 0.005 | 3 [-1;6] | | 0.11 | 810 | 3 [1;6] | 0.012 | 2 [-2;5] | 0.31 |
| Insulin**^§,¶^**, pmol/L | 746 | -9 [-14;-5] | | | <0.001 | -28 [-33;-22] | | <0.001 | 743 | -10 [-14;-5] | <0.001 | -26 [-32;-21] | <0.001 |
| HDL cholesterol, nmol/L | 791 | 0.1 [-0.01;0.1] | | | 0.10 | 0.2 [0.1;0.3] | | <0.001 | 788 | 0.1 [-0.01;0.1] | 0.08 | 0.2 [0.1;0.3] | <0.001 |
| LDL cholesterol, nmol/L | 791 | -0.01 [-0.1;0.1] | | | 0.89 | -0.2 [-0.4;-0.1] | | 0.004 | 788 | -0.02 [-0.2;0.1] | 0.80 | -0.3 [-0.4;-0.1] | 0.003 |
| TAG**^§^**, mmol/L | 791 | -0.02 [-0.08;0.03] | | | 0.43 | -0.15 [-0.22;-0.09] | | <0.001 | 788 | -0.02 [-0.07;0.03] | 0.44 | -0.14 [-0.20;-0.07] | <0.001 |

UW underweight, NW normalweight, OW overweight/obesity, CI confidence interval, BMC bone mineral content, BMD bone mineral density, BA bone area, IGF-I insulin-like growth factor-1, TAG triacylglycerol, E% percentage of energy intake, MJ megajoules
**^†^**Data are presented as regression coefficients ($\beta$) with 95% confidence intervals (CI) and *P* values derived from unadjusted linear regression models
**^‡^**Data are presented as regression coefficients ($\beta$) with 95% confidence intervals (CI) and *P* values derived from linear mixed-effects models with weight group, sex, age (continuous), height (continuous) and parental education level as fixed effects and class and school as random effects
**^§^**Dependent variable was ln-transformed to meet model assumptions and the table presents back-transformed estimates on the original scale based on the method by Laursen et al. [5]
**^¶^**The adjusted analysis did not include height as fixed effect
**^††^**Analysis conducted only among eaters of fish, i.e. children with weekly fish intake > 0 g/d

**REFERENCES**

1. Cole TJ, Lobstein T (2012) Extended international (IOTF) body mass index cut-offs for thinness, overweight and obesity: Extended international BMI cut-offs. Pediatr Obes 7(4):284–94. doi:10.1111/j.2047-6310.2012.00064.x

2. Cole TJ, Bellizzi MC, Flegal KM et al (2000) Establishing a standard definition for child overweight and obesity worldwide: international survey. BMJ 6;320(7244):1240–1240. doi:10.1136/bmj.320.7244.1240

3. Cole TJ, Flegal KM, Nicholls D et al (2007) Body mass index cut offs to define thinness in children and adolescents: international survey. BMJ 28;335(7612):194. doi:10.1136/bmj.39238.399444.55

4. Blomhoff R, Andersen R, Arnesen EK et al (2023) Nordic Nutrition Recommendations 2023. Copenhagen: Nordic Council of Ministers. https://pub.norden.org/nord2023-003. Accessed 4 March 2024

5. Laursen RP, Dalskov SM, Damsgaard CT, Ritz C (2014) Back-transformation of treatment differences—an approximate method. Eur J Clin Nutr 68(2):277–80. doi:10.1038/ejcn.2013.259
